# Genetic liability to asthma and risk of cardiovascular diseases: A Mendelian randomization study

**DOI:** 10.3389/fgene.2022.879468

**Published:** 2022-07-26

**Authors:** Heng Chen, Wei Chen, Liangrong Zheng

**Affiliations:** ^1^ Department of Cardiology, The First Affiliated Hospital, College of Medicine, Zhejiang University, Hangzhou, China; ^2^ Department of Respiratory and Critical Care Medicine, Ruian People’s Hospital, Wenzhou, China

**Keywords:** Mendelian randomization, causal association, asthma, atrial fibrillation, heart failure, coronary artery disease, stroke

## Abstract

**Background and Aims:** Epidemiological studies have suggested positive associations between asthma and the risk of cardiovascular diseases (CVDs). However, causality remains inconclusive. We aim to explore the causal associations between asthma and CVDs risk using the Mendelian Randomization (MR) approach.

**Methods:** We obtained summary-level data for eight CVDs [including atrial fibrillation (AF), coronary artery disease (CAD), heart failure (HF), stroke, ischemic stroke, large artery stroke, small vessel stroke, and cardioembolic stroke] from several large genome-wide association studies (GWASs) and the FinnGen consortium. Nine lead single-nucleotide polymorphisms associated with asthma (*p* < 5 × 10^−8^) were identified from the GWAS conducted by the Trans-National Asthma Genetic Consortium. MR analyses were performed using the inverse variance weighted method, supplemented by the weighted median and MR-Egger methods.

**Results:** Inverse variance weighted method showed suggestive effects of genetically determined asthma on AF (odds ratio (OR), 1.08; 95% confidence interval (CI), 1.02, 1.14; *p* = 0.009) and HF (OR, 1.05; 95% CI, 1.01, 1.09; *p* = 0.029). We found no causal associations between asthma and other CVDs. No horizontal pleiotropy was observed.

**Conclusion:** This MR study provides genetic evidence suggesting a causal association between asthma and the risk of AF and HF, although not at the level of significance after multiple testing correction. Programs aimed at treating asthma among asthmatics might help prevent the adverse health effects inflicted by CVDs.

## Introduction

With the aging of the global population, cardiovascular diseases (CVDs) have become major public health problems and represent the leading causes of morbidity and mortality worldwide (Lennon et al., 2018). Primary prevention strategies for CVDs have been extensively studied for decades.

Asthma is a common respiratory disorder characterized by reversible airflow obstruction (Moorman et al., 2012). Epidemiological studies have reported associations between asthma and CVDs. A cohort study including 54,567 individuals showed that patients with asthma experienced a higher risk of atrial fibrillation (AF) (adjusted hazard ratio (HR), 1.38, 95% CI, 1.18, 1.61) over a 15.4-years follow-up (Cepelis et al., 2018). A meta-analysis demonstrated that asthma was associated with increased risks of coronary artery disease (CAD) in both prospective studies (HR, 1.34, 95% CI, 1.09, 1.64) and retrospective studies (odds ratio (OR), 1.29, 95% CI, 1.13, 1.46) (Wang et al., 2017). Besides, asthma has been associated with a higher risk of heart failure (HF) as well as stroke (Iribarren et al., 2012; Wen et al., 2016). However, causality remains unknown. It is uncertain whether previously observed associations were biased by residual confounders and/or reverse causation (Smith and Ebrahim, 2003).

Mendelian Randomization (MR) is an extensively used approach to investigated causal relationships between exposures and outcomes. It takes advantage of naturally randomized genetic variants as instrumental variables (IVs), and will not be easily affected by confounding factors or reverse causation (Lawlor et al., 2008). A very recent MR study conducted by Zhou et al. explored a similar topic (Zhou et al., 2021). In the present study, we independently used different IVs and different data sources for CAD, MI and HF. Importantly, for the first time we investigated the potential causal association between asthma and AF.

## Methods

### Study design

We performed a two-sample MR study to investigate the causal associations between asthma and the risk of CVDs. The MR approach was based on three key assumptions. First, IVs should be robustly associated with CVDs; second, there is no association between the SNPs and potential confounders; third, IVs lead to CVDs only through their effects on asthma (Burgess et al., 2015). A checklist of the Strengthening the Reporting of Observational Studies in Epidemiology Using Mendelian Randomization (STROBE-MR) guideline (Skrivankova et al., 2021) was provided in Supplementary Table S1.

### Outcome data sources

Detailed information on data sources is presented in Table 1. We included eight CVDs endpoints with cases numbers ranging from 4,373 (large artery stroke) to 60,801 (coronary artery disease). Summary statistics for the associations of the IVs with CVDs were obtained from Coronary ARtery DIsease Genome-wide Replication and Meta-analysis plus The Coronary Artery Disease Genetics (CARDIoGRAMplusC4D) consortium for CAD (Nikpay et al., 2015), GWAS meta-analysis by Nielsen et al. for AF (Nielsen et al., 2018), Heart Failure Molecular Epidemiology for Therapeutic Targets (HERMES) consortium for HF (Shah et al., 2020), and MEGASTROKE consortium for stroke and stroke subtypes (Malik et al., 2018). We also used summary-level data from FinnGen (FinnGen_Consortium, 2022) for replication purpose. FinnGen is a medical project launched in 2017 that includes 500,000 Finnish participants and aims to combine genomic information with health data so as to improve human well-being through genetic research (FinnGen_Consortium, 2022). Ethics approval and informed consent were provided in these publicly available databases.

**TABLE 1:** Detailed information of studies and datasets used for analyses.

Phenotype	Data source	Cases	Controls	Population
Asthma	TAGC	19,954	107,715	European
Atrial fibrillation	Nielsen et al	60,620	970,216	European
	FinnGen	28,670	135,821	European
Coronary artery disease	CARDIoGRAMplusC4D	60,801	123,504	77% European
	FinnGen	25,707	234,698	European
Heart failure	HERMES	47,309	930,014	European
	FinnGen	30,098	229,612	European
Stroke	MEGASTROKE	40,585	406,111	European
	FinnGen	22,791	190,836	European
Ischemic stroke	MEGASTROKE	34,217	406,111	European
	FinnGen	12,948	10,551	European
Large artery stroke	MEGASTROKE	4,373	146,392	European
Small vessel stroke	MEGASTROKE	5,386	192,662	European
Cardioembolic stroke	MEGASTROKE	7,193	204,570	European

TAGC, Trans-National Asthma Genetic Consortium; CARDIoGRAMplusC4D, Coronary ARtery DIsease Genome-wide Replication and Meta-analysis (CARDIoGRAM) plus The Coronary Artery Disease (C4D) genetics; HERMES, heart failure molecular epidemiology for therapeutic targets.

### Validation of instrumental SNPs

First, we identified 16 single-nucleotide polymorphisms (SNPs) associated with asthma at the genome-wide significance level (*p* < 5 × 10^−8^) from a large GWAS meta-analysis conducted by the Trans-National Asthma Genetic Consortium (included up to 19,954 cases and 107,715 controls of European descent) (Demenais et al., 2018) (Table 1). Asthma cases were defined based on clinical diagnosis and/or standardized questionnaires. Approximately 37% of cases were defined as asthma onset at or before 16 years of age and the rest were defined as adult asthma (Demenais et al., 2018). Valid MR estimates depended on no linkage disequilibrium (*r* ^2^ < 0.001) across selected SNPs; thus, we removed rs11071558 since it was in correlation with other SNPs (based on 1000 genomes project, Phase 3) (Abecasis et al., 2010). The 1000 Genomes Project used samples from self-reported healthy people and provided a catalogue of human genome sequence variation (Abecasis et al., 2010). Then, we searched for SNPs with possible horizontal pleiotropic effects in Phenoscanner V2 (Kamat et al., 2019). A confounder indicates a biological pathway through which IVs can affect the outcome rather than through the exposure, leading to bias in MR estimates. We removed SNPs that were strongly associated (*p* < 5 × 10^−8^) with CVDs risk factors (confounders) such as hypertension, hypothyroidism and diabetes (Supplementary Table S2). To meet the third assumption, a SNP (rs2033784) that were significantly associated (*p* < 5 × 10^−8^) with the outcome (CAD) directly were also dropped. Finally, nine asthma-associated SNPs were leveraged as IVs (Supplementary Table S3). Furthermore, F-statistics were calculated to detect the presence of weak IVs bias with the following formula: F=*R* ^2^ × (N-2)/(1-R^2^) (Burgess and Thompson, 2011), where *R* ^2^ indicates the proportion of variance in asthma explained by each selected SNP (calculated with the method described previously (Shim et al., 2015)) and N represents the sample size. No proxy-SNP was necessary since all the SNPs that we used were available in the CVDs datasets.

### Statistical analysis

Wald estimator was used to calculate MR estimates. We employed the inverse variance weighted (IVW) (Burgess et al., 2017) approach in the multiplicative random-effects model as the main statistical method to assess the causal associations between genetic liability to asthma and the risk of CVDs. Then, we combined MR estimates for each outcome from different data sources using the fixed-effects meta-analysis method. The IVW method provides the highest statistical power but may be biased if IVs exhibit horizontal pleiotropy (Bowden et al., 2015). Therefore, we performed sensitivity analyses including the weighted median (Bowden et al., 2016) and the MR-Egger regression (Burgess and Thompson, 2017) methods to test the robustness of our results and potential pleiotropy. The weighted median method can provide unbiased causal estimates when more than half of the weight comes from valid SNPs (Bowden et al., 2016). MR-Egger regression method can generate estimates corrected for pleiotropy (Burgess and Thompson, 2017). In addition, a *p*-value < 0.05 for the MR-Egger intercept was considered to indicate the presence of pleiotropic bias (Bowden et al., 2015). Furthermore, we calculated the I^2^ statistics to assess the degree of heterogeneity among the SNPs in the IVW analyses (low, >25%; moderate, >50%; and high, >75%) (Greco M et al., 2015) (Supplementary Table S4). The OR estimate of CVDs and the corresponding confidence intervals (CIs) were scaled per-1-log unit increase in the risk of asthma. A two-sided *p*-value of <0.0063 (0.05/8 outcomes) was considered statistically significant, and associations with *p*-values between 0.0063 and 0.05 were deemed as suggestive associations. All MR analyses were conducted using the TwoSampleMR package in R Software 4.1.0 (R_Core_Team, 2021).

## Results

Detailed information on the characteristics of the selected SNPs is provided in Supplementary Table S3. Altogether they explained approximately 5.22% of the phenotypic variability of asthma. All the included SNPs have F-statistics higher than 30, suggesting that they were strong enough to predict asthma in the present study. There is no sample overlap between the data sources for asthma and CVDs.

**FIGURE 1:**
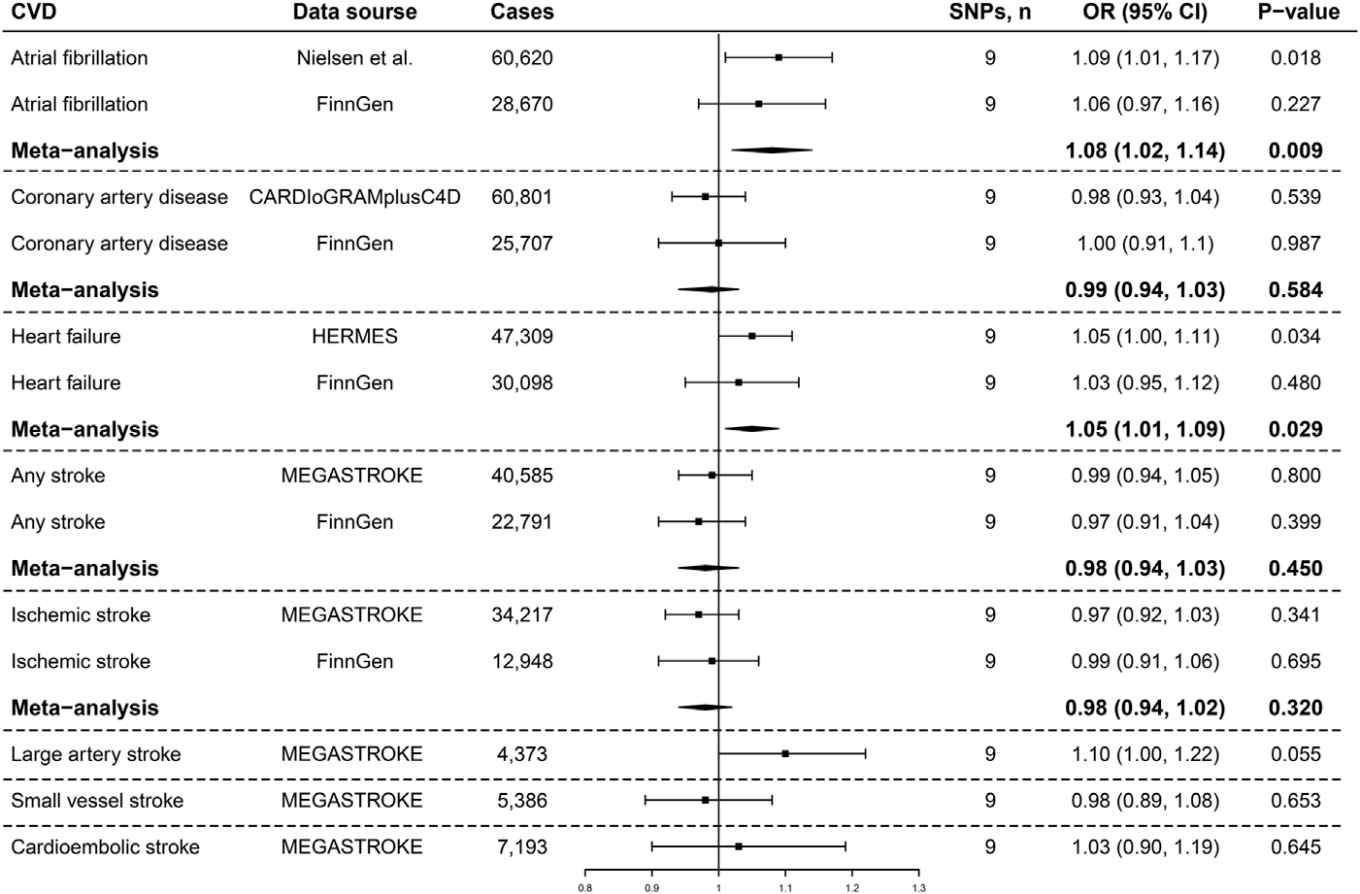
Associations of genetic liability to asthma with the risk of cardiovascular diseases. SNPs, single-nucleotide polymorphisms; OR, odds ratio; CI, confidence interval; CARDIoGRAMplusC4D, Coronary ARtery DIsease Genome-wide Replication and Meta-analysis plus The Coronary Artery Disease Genetics; HERMES, Heart Failure Molecular Epidemiology for Therapeutic Targets.

IVW analyses showed suggestive causal associations between genetically determined asthma and the risk of AF in the GWAS meta-analysis conducted by Nielsen et al. (OR, 1.09; 95% CI, 1.01, 1.14; *p* = 0.018; Figure 1) and HF in HERMES (OR, 1.05; 95% CI, 1.00, 1.11; *p* = 0.034; Figure 1). Both the associations remained directionally consistent in the FinnGen consortia, and the suggestive associations persisted in the meta-analysis combining different data sources (AF: OR, 1.08; 95% CI, 1.02, 1.14; *p* = 0.009; HF: OR, 1.05; 95% CI, 1.01, 1.09; *p* = 0.029; Figure 1). We did not observe a clear pattern of associations between genetic liability to asthma and the risk of CAD, stroke, or stroke subtypes (Figure 1).

The associations of asthma with AF and HF were directionally consistent but with noticeably wider CIs in sensitivity analyses based on the weighted median and MR-Egger methods (Supplementary Table S4). The results of no associations between genetically predicted asthma and other CVD outcomes were replicated in both sensitivity analyses (Supplementary Table S4). We found modest heterogeneity in several analyses. For all outcomes considered, there was no evidence of horizontal pleiotropy based on the intercept term in the MR-Egger regression (*P*_intercept_ > 0.05) (Supplementary Table S4). Scatter plots of the SNP-exposure and SNP-outcome associations were provided in Supplementary Figures S1–S4.

## Discussion

This MR study suggests causal associations between genetically determined asthma and the risk of AF and HF. No clear pattern of associations of asthma with the risk of CAD, stroke, or stroke subtypes were found.

Given that asthma and AF shared an underlying inflammatory pathophysiological mechanism (Busse, 1998; Andrade et al., 2014), investigators in the past decade have sought to assess the association between asthma and AF risk using epidemiological approaches. A population-based nested case-control study suggested asthma to be associated with an increased risk of AF (OR 1.2; 95% CI 1.11–1.30) (Chan et al., 2014). In a large multiethnic cohort study (included 17,514 individuals) with 12.9 years of follow-up, the researchers reported that it was patients with persistent asthma, rather than those with intermittent asthma, who experienced a higher risk of atrial fibrillation (hazard ratio (HR), 1.49; 95% CI, 1.03–2.14) compared with non-asthmatics (Tattersall et al., 2020). The association was further strengthened by another prospective population cohort study that revealed a dose-response relationship between levels of asthma control and the risk of AF (Cepelis et al., 2018). In line with these findings, this MR study provides genetic evidence showing that asthma may lead to an increased risk of AF. Medication use for asthma control, such as β2-agonists, is one of the potential explanations for the causal association; the drug has been demonstrated to increase the risk of arrhythmias (Salpeter et al., 2004). Besides, chronic inflammation in the pathogenesis of asthma may cause electrical and structural remodeling of the atria, thereby participating in the onset and continuation of AF (Murdoch and Lloyd, 2010; Hu et al., 2015).

Our result regarding the suggestive causal association between asthma and the risk of HF collaborates with a prospective cohort study which reported that asthma was associated with a 114% increased risk of HF (Iribarren et al., 2012). On the other hand, Sun et al. (Sun et al., 2017) performed prospective analyses among 1118 participants with or without a history of asthma from childhood. They reported that adults with a history of childhood asthma had a greater left ventricular mass index, which was commonly used as a predictor for progression and severity stages of HF (Shah et al., 2017). However, mechanisms for this epidemiological association remain unestablished. Given that AF is one of the well-known risk factors for HF (Carlisle et al., 2019), we recognize that the asthma-AF association may, at least in part, explain the asthma-HF association that we observed.

Data surrounding the association between asthma and CAD is controversial among prospective studies and retrospective studies (Liss et al., 2000; Iribarren et al., 2004; Onufrak et al., 2008; Iribarren et al., 2012). To determine whether asthma leads to a higher risk of CAD, Liu et al. conducted a meta-analysis that included seven studies with 12 cohorts (Liu et al., 2017). The analysis concluded that there was a significant relationship between asthma and CAD risk (HR, 1.42; 95% CI, 1.30–1.57) (Liu et al., 2017). Likewise, epidemiological evidence from another meta-analysis suggested a significant association of asthma with stroke risk (HR, 1.32; 95% CI, 1.13–1.54) (Wen et al., 2016). Our study, on the contrary, does not provide evidence of causal effects of asthma on CAD, stroke, or stroke subtypes, suggesting that associations observed clinically are likely to be biased. Further clinical study on a larger scale is warranted to better elucidate this issue.

A recent MR study on the similar topic reported by Zhou et al. (Zhou et al., 2021) shared some conclusions with us, including the causal association between asthma and HF, and no causal associations for CAD, stroke, and stroke subtypes. Their independent study used different IVs and came to similar results. However, AF was included as an outcome in the present study for the first time. Our analyses further revealed that efforts to treat asthma will probably lead to a reduced risk of AF.

The major strength of this study is the MR method which is less susceptible to potential confounding factors and other biases, thus reinforcing the causal inference. Second, we assessed the potential associations using summary statistics from several large GWASs. The similar results across different data sources assured the reliability of our findings. In addition, our findings were unlikely to be impacted by population structure bias since the analyses were restricted to individuals of European ancestry, except for the analysis for CAD (77% European; Table 1).

There are several limitations worth noting in this study. First, although we have excluded potential pleiotropic SNPs and MR-Egger regression indicated little horizontal pleiotropy, the influence of pleiotropy bias remains a concern in this study. Specifically, psychiatric traits such as major depressive disorder and neuroticism may be confounding factors in the causal associations between asthma and CVDs (Cao et al., 2021; Wu et al., 2021; Zhang et al., 2021, 2022). Besides, the divergent results of sensitivity analyses may indicate a bias of genetic pleiotropy towards asthma-AF and asthma-HF associations that we observed. Second, we are not able to detect potential non-linear associations between asthma severity and the risk of CVDs since this study was based on summary-level data. In addition, the mixed population of CardiogramplusC4D consortium may affect the result of the corresponding analysis. Therefore, caution must be applied here. Another limitation is the limited generalizability of this study given the restriction of subjects largely of European ancestry. It would be of great interest to explore the causal associations on other populations. Future studies are warranted to extend our findings.

## Conclusion

This MR study revealed suggestive causal associations between asthma and the risk of AF and HF, despite none of the associations achieve a significance value upon multiple testing correction either in the original analysis or the meta-analysis. Interventions to treat asthma might benefit the primary prevention for AF and HF among asthmatics. No causal associations were observed between asthma and the risk of CAD, stroke, or stroke subtypes.

## Data Availability

The original contributions presented in the study are included in the article/Supplementary Material, further inquiries can be directed to the corresponding author.
