# Supplementary material for: Genetic liability to asthma and risk of cardiovascular diseases: A Mendelian randomization study
Source: Front Genet. 2022 Jul 26;13:879468. doi: 10.3389/fgene.2022.879468 (PMC9360591; doi:10.3389/fgene.2022.879468)
Supplement: Supplementary file 1 [file DataSheet1.docx]

Supplementary Figure

**Supplementary Figure 1**


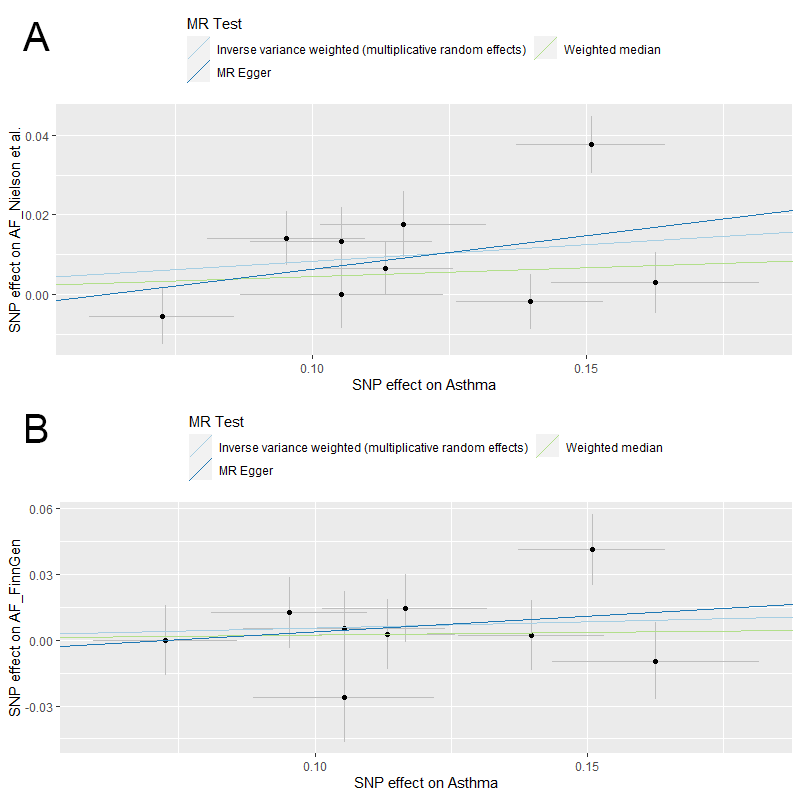


**Supplementary Figure 1.** Scatter plot of SNP-Asthma and SNP-AF associations in Neilson et al. GWAS (A) and FinnGen dataset (B).

AF, atrial fibrillation.

**Supplementary Figure 2**


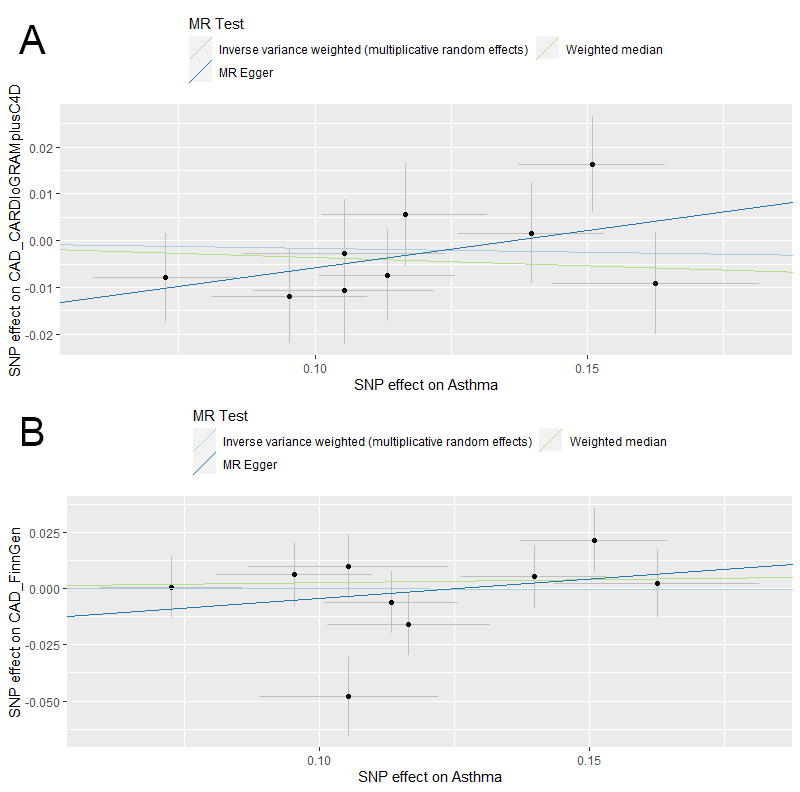


**Supplementary Figure 2.** Scatter plot of SNP-Asthma and SNP-CAD associations in (A) and FinnGen dataset (B).

CAD, coronary artery disease; CARDIoGRAMplusC4D, Coronary ARtery DIsease Genome-wide Replication and Meta-analysis plus The Coronary Artery Disease Genetics.

**Supplementary Figure 3**


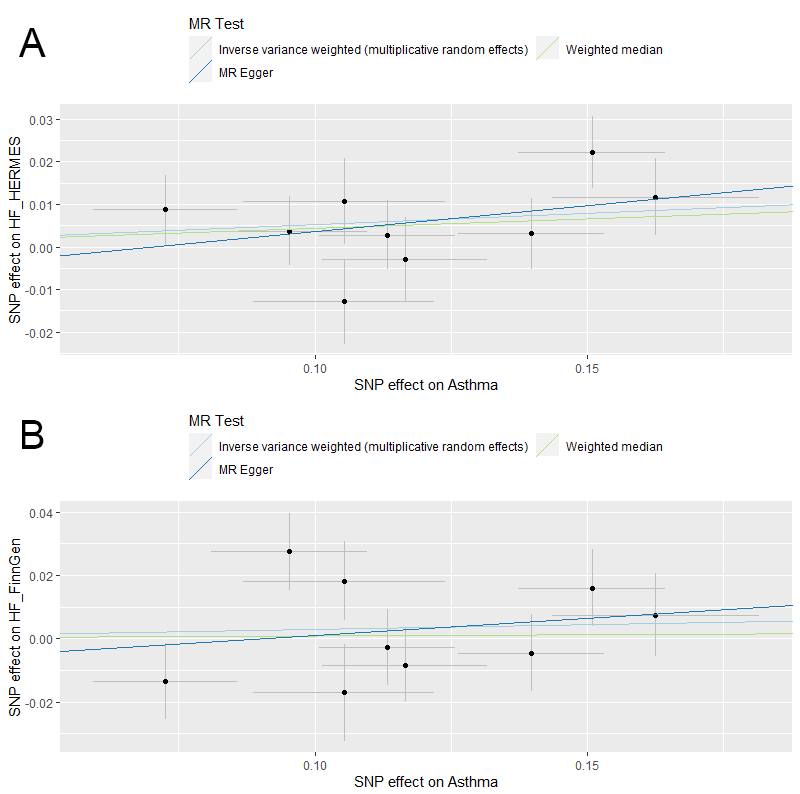


**Supplementary Figure 3.** Scatter plot of SNP-Asthma and SNP-HF associations in HERMES (A) and FinnGen dataset (B).

HF, heart failure; HERMES, Heart Failure Molecular Epidemiology for Therapeutic Targets.

**Supplementary Figure 4**


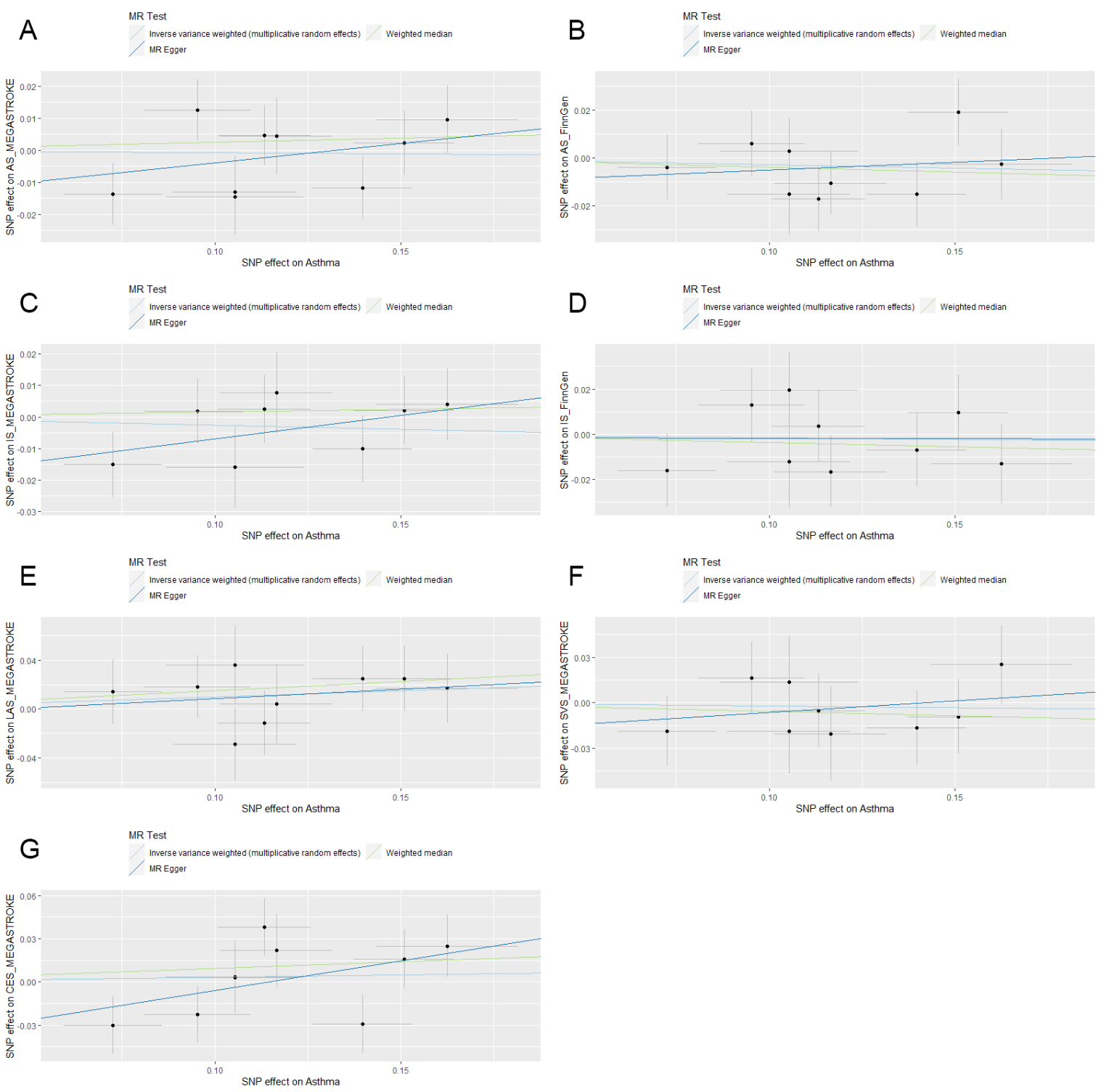


**Supplementary Figure 4.** (A-B) Scatter plot of SNP-Asthma and SNP-AS associations in MEGASTROKE and FinnGen dataset; (C-D) Scatter plot of SNP-Asthma and SNP-IS associations in MEGASTROKE and FinnGen dataset; (E) Scatter plot of SNP-Asthma and SNP-LAS associations in MEGASTROKE; (F) Scatter plot of SNP-Asthma and SNP-SVS associations in MEGASTROKE; (G) Scatter plot of SNP-Asthma and SNP-CES associations in MEGASTROKE.

AS, any stroke; IS, ischemic stroke; LAS, large artery stroke; SVS, small vessel stroke; CES, cardioembolic stroke.
